# A Framework of Visual Checkout System Using Convolutional Neural Networks for Bento Buffet

**DOI:** 10.3390/s21082627

**Published:** 2021-04-08

**Authors:** Mei-Yi Wu, Jia-Hong Lee, Chuan-Ying Hsueh

**Affiliations:** 1 Graduate Institute of Food Culture and Innovation, National Kaohsiung University of Hospitality and Tourism, Songhe Road, Xiaogang District, Kaohsiung City 812, Taiwan; 2 Department of Information Management, National Kaohsiung University of Science and Technology, Songhe Road, Xiaogang District, Kaohsiung City 807, Taiwan; jhlee@nkust.edu.tw (J.-H.L.); f107118109@nkust.edu.tw (C.-Y.H.)

**Keywords:** food image recognition, convolutional neural network, Bento buffet, visual checkout system, food plate detection, food volume, depth image

## Abstract

In recent years, the technology of artificial intelligence (AI) and robots is rapidly spreading to countries around the world. More and more scholars and industry experts have proposed AI deep learning models and methods to solve human life problems and improve work efficiency. Modern people’s lives are very busy, which led us to investigate whether the demand for Bento buffet cafeterias has gradually increased in Taiwan. However, when eating at a buffet in a cafeteria, people often encounter two problems. The first problem is that customers need to queue up to check out after they have selected and filled their dishes from the buffet. However, it always takes too much time waiting, especially at lunch or dinner time. The second problem is sometimes customers question the charges calculated by cafeteria staff, claiming they are too expensive at the checkout counter. Therefore, it is necessary to develop an AI-enabled checkout system. The AI-enabled self-checkout system will help the Bento buffet cafeterias reduce long lineups without the need to add additional workers. In this paper, we used computer vision and deep-learning technology to design and implement an AI-enabled checkout system for Bento buffet cafeterias. The prototype contains an angle steel shelf, a Kinect camera, a light source, and a desktop computer. Six baseline convolutional neural networks were applied for comparison on food recognition. In our experiments, there were 22 different food categories in a Bento buffet cafeteria employed. Experimental results show that the inception_v4 model can achieve the highest average validation accuracy of 99.11% on food recognition, but it requires the most training and recognition time. AlexNet model achieves a 94.5% accuracy and requires the least training time and recognition time. We propose a hierarchical approach with two stages to achieve good performance in both the recognition accuracy rate and the required training and recognition time. The approach is designed to perform the first step of identification and the second step of recognizing similar food images, respectively. Experimental results show that the proposed approach can achieve a 96.3% accuracy rate on our test dataset and required very little recognition time for input images. In addition, food volumes could be estimated using the depth images captured by the Kinect camera, and a framework of visual checkout system was successfully built.

## 1. Introduction

With the rapid spread of artificial intelligence (AI) and smart sensor technology to countries around the world, more and more scholars and experts in the field have proposed a variety of deep learning models and algorithms to solve human life problems and improve their effectiveness. One of the most high-profile examples in the retail industry is the development of unmanned stores. The first Amazon Go was successfully launched in Boston in 2016. Amazon Go is an unmanned supermarket with a fast checkout experience of “Just Walk Out Shopping.” The shopping model in which consumers can check out without the line has become the hottest topic. Discuss the reasons why unmanned stores can drive the market. On the one hand, e-commerce operators try to expand the market and connect the physical retail field. On the other hand, traditional retailers hope to reduce their demand for manpower. Amazon Go combines computer visual recognition technology in the store with a variety of environmental sensors to realize a fully automated unmanned self-checkout system.

**Figure 1:**
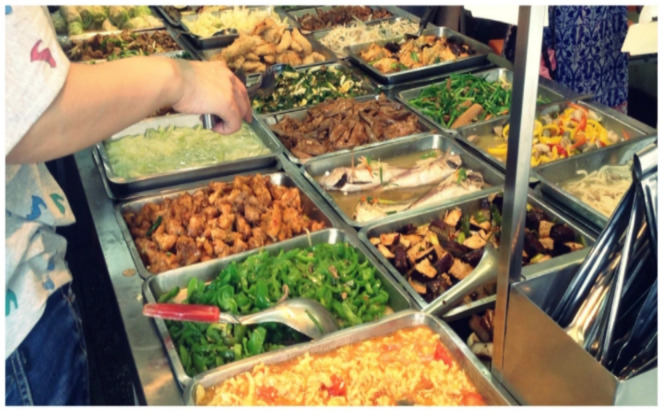
Foods in a Bento buffet cafeteria.

**Figure 2:**
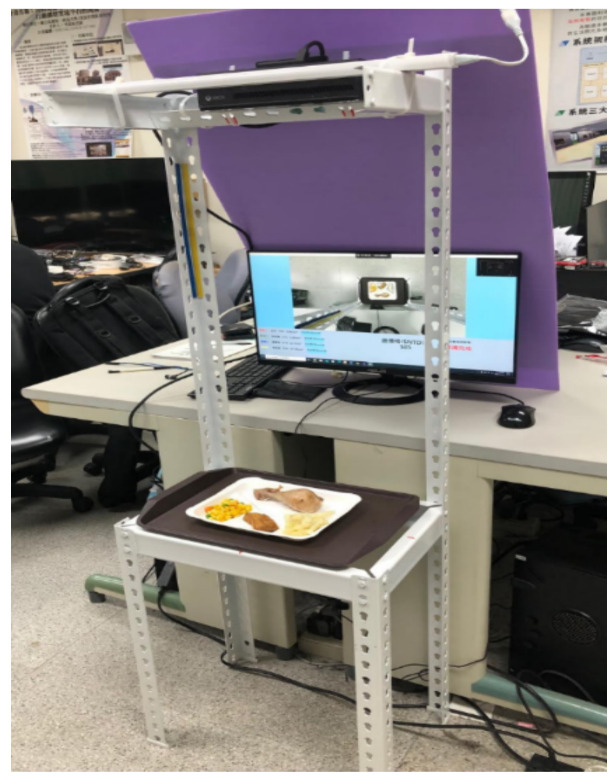
The proposed prototype for food recognition and pricing.

The most typical Taiwan meal is the Bento buffet, and the cafeterias can be found everywhere. It originated from the Japanese Bento and is a staple in Taiwan cuisine. When a customer walks into a Bento buffet cafeteria, he or she must take an empty food plate first and then pick food from a buffet-style counter that is filled with plates of meat and vegetables. Figure 1 shows the inside of a Bento buffet cafeteria. However, the customer has to pay for everything that he or she put on the plate, and the price of dishes is usually calculated by a cashier of the restaurant. The customer always being told to pay a price for the dishes taken instead of a proper listing on the price of each dish. This process sometimes makes the customers remain uncertain or feel unfair about the calculated price by the cashier. In addition, Taiwanese people are accustomed to dining in cafeterias at noon or in the evening, but dining at peak times usually takes too much time in line for the cashier to calculate the prices of food. Therefore, an automatic food price system is required to reduce the waiting time of customers. In this paper, we proposed a possible solution to overcome the above problems using food image recognition and food volume estimation. The study aims to train a convolutional neural network (CNN) model to recognize dishes from the food images and estimate the food volumes using depth images of dishes. We built a prototype of the Bento buffet pricing system using an angle steel shelf, a Kinect camera, a light source, and a desktop computer. Figure 2 shows the prototype of the system structure.

## 2. Literature Review

**Figure 3:**
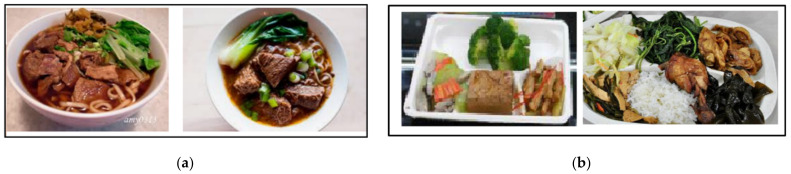
Two different categories of food—(**a**) single dish, beef noodle and (**b**) mixed food, buffet.

Different kinds of food are cooked according to the cultures and environments. Food plays an important role in daily life. Recently, many research studies on food image recognition have been proposed. Chinese food image classification is a great challenge since food images from the same category are captured with different patterns, shapes, and perspectives, according to the people who take the images. Food images can be roughly divided into two categories. The first category is a single dish, such as beef noodles, braised pork rice, and stinky tofu. Each food has its own individual container to hold. Therefore, the entire image can be used as input for food image recognition. Usually, there is no need for preprocessing of detailed image cutting. The other type is mixed dishes, such as a buffet. This type of food is usually placed on the food plate. Therefore, when performing food identification, the image segmentation of each dish on the plate must be performed first. The identification of mixed food is generally considered a difficult problem. The main challenge is that if different dishes on a plate are not neatly arranged, they may overlap each other, and there may be no clear boundaries between them. For food image identification, the entire plate image must be segmented first, and various dishes are cut out from the image. The overall food image identification is more difficult and cumbersome than ordinary object identification. An example of these two types of food images is shown in Figure 3.

Many research papers in the literature address food classification as a unique pattern recognition problem. Generally, food image recognition can be divided into two ways—the handcraft-feature approach and deep-learning approaches. The term “handscraft feature” comes from the researcher’s ability to identify relevant features of a particular object in an image. When classifying food, the shape, color, and texture of the food will be different. The selection of relevant features must be related to these three aspects [1]. Thus far, challenges remain when it comes to identifying prepared food. Different food preparation methods may lead to different characteristics [2]. For example, the ingredients of a prepared salad and the shape and appearance of the whole fruit or vegetable have different shapes and textures. In order to determine the best feature extraction process, informative visual data must be extracted from food images. Commonly used features mainly use the color, shape, or texture calculation of the image, and represent the image through the calculated statistical distribution or transformed into a feature vector. In the feature-based food image recognition, we divide the relevant process into three steps—the establishment of a food image database, the calculation of food image features, and the image classification method. Commonly used image features include scale-invariant feature transform (SIFT) [3], bag of features (BoF) [4], local binary pattern (LBP) [5], etc., and commonly used image classification methods include k-dimensional tree (KD tree) [6], k-nearest neighbors algorithm (KNN) [7], support vector machine (SVM) [8], or back propagation neural network (BPNN) [9], etc.

Deep learning is part of machine learning, a new method of learning and training more effective neural networks. The built-in mechanism of the deep-learning algorithm passes a series of connected layers, and the final one is responsible for classification, i.e., the fully connected layer, which automatically uses feature extraction. Compared with other traditional methods, it has better performance and enhanced processing power. These methods usually must be trained using large datasets but have excellent classification capabilities. Convolutional Neural Network (CNN) is one of the most outstanding techniques in deep learning. Since CNN has an excellent ability to learn visual data and obtain high precision for challenging tasks with large-scale image data, it is widely used in computer vision applications [10]. Compared with other traditional image feature extraction methods, CNN performs much better. Bossard et al. [11] implemented the CNN model based on the previously proposed network architecture [12,13]. Using images from the Food-101 dataset, an average accuracy rate of 56.4% was obtained in 450,000 iterations. Yanai and Kawano [14] implemented a deep convolutional neural network (DCNN) on three different food datasets Food-101, UEC-FOOD-100, and UECFOOD-256.

Pandey et al. [15] believe that different convolutional neural networks can extract different image features, and therefore, they proposed to use three CNN convolutional neural networks (AlexNet, GoogLeNet, ResNet) to train the features and weights, respectively, and finally combine the trained features to perform classification. Their experimental results show that when the FOOD-101 database was used, a single CNN performs better with ResNet, with an accuracy rate of 67.6%, while using three CNNs at the same time can achieve maximum accuracy of 72.1%. According to the survey by Subhi et al. [16], the effectiveness of pretraining and fine-tuning DCNN with 100 pieces of each food category obtained from each dataset as training images.

In this paper, our main contribution is the use of state-of-the-art deep convolutional neural networks to solve the need for food automatic recognition in Taiwan’s Bento buffet cafeterias. A prototype including an angle steel shelf, Kinect camera, and LED light was created. In this approach, we capture a large amount of food from one cooperated cafeteria and use depth images to estimate the food volumes. Finally, we have successfully complemented a Bento buffet pricing system. The paper is organized as follows: In Section 2, the literature review is presented. In Section 3, the proposed method is described. Experimental results are reported in Section 4. The last section is the conclusion and future research.

## 3. Proposed Methods

### 3.1. System Flowchart

**Figure 4:**
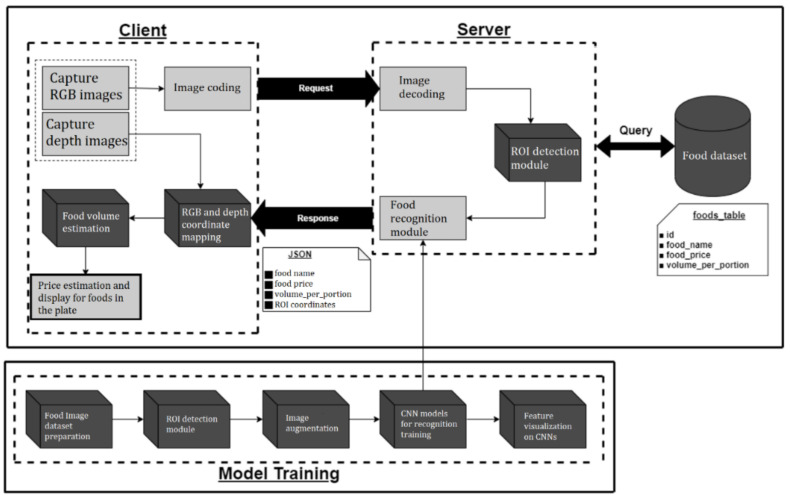
The system architecture of the proposed method.

Figure 4 shows the flowchart of the proposed food price estimation framework. It consists of two stages—the training stage and the test stage. In the training stage, the following major steps are executed:

1. Put food on an empty plate and place it on the shelf;
2. Take a photo and obtain an RGB image;
3. Detect the regions of interest (ROIs) and cut the individual food images and then build the food image dataset;
4. Apply the food image dataset to train the parameters of CNN models.

In the test stage, the following steps are executed:

1. Put food on an empty plate and place it on the shelf;
2. Take a photo to obtain an RGB image and a depth image;
3. Detect ROIs and cut the individual food images for testing;
4. Perform food recognition on test images using the trained CNN models;
5. Estimate the food volumes using the depth image;
6. Sum up the price for each food on the food plate and display it on the screen.

The detailed operations for major steps are described in the following sections.

### 3.2. Image Preprocessing and ROI Detection

**Figure 5:**
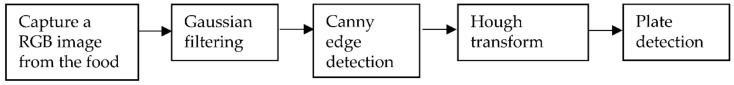
The plate detection using Hough transformation.

In this study, we set up the food plate as the region of interest (ROI). Plate detection is a critical operation to automatically capture the food images from the plate. A straightforward method to detect ROI in images is using Hough line transformation. If we can obtain the four straight lines surrounding the food plate, then the four corners of the plate and its bounding box are determined. Hough’s working method is basically to scan the entire image and use a transformation to convert all white pixels from Cartesian coordinates to polar coordinates; black pixels are ignored. Therefore, it is necessary to detect the edges well before performing Hough transform; otherwise, its efficiency will be further reduced. The plate detection process is shown in Figure 5.

In addition, noisy images will not allow Hough transform to achieve good results since noises will cause erroneous white pixels in edge detection.

Before the plate detection process, Gaussian smoothing [16] with an r×r kernel was performed to remove noises in the captured images. The general mathematical expression of this filter used to eliminate the noises on the image is presented in Equation (1).

(1) $$G\left(x,y\right)=\frac{1}{2\pi \sigma^{2}}e^{-\left(x^{2}+y^{2}\right)/2\sigma^{2}},$$

where *r* is the radius $r^{2}=x^{2}+y^{2}$, σ is the standard deviation of the distribution, and *x* and *y* are the location indices. In our experiments, we set r=3 and σ=1.

**Figure 6:**
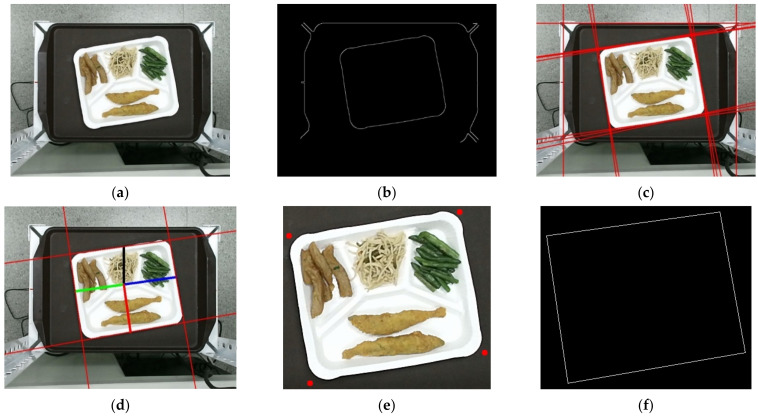
The process of food plate region detection: (**a**) original test image; **(b**) edge image; (**c**) detected lines by Hough transformation; (**d**) the closed four line segments surrounding the food plate; (**e**) four corner points; (**f**) the bounding box of the food plate.

Then, Canny edge detection [17] was applied to obtain a binary edge image shown in Figure 6b. The edge image was used as input for Hough transform [18] to detect line segments. The Hough transform is perhaps most often used for detecting line segments in edge maps. A line segment in 2D can be described with two real-valued parameters using the classic slope-intercept form

(2) y=kx+d,

where *k* is the slope and *d* the intercept, that is, the height at which the line would intercept the *y* axis. A line segment that passes through two given edge points. Here, we have set a threshold of line length to leverage possible straight lines which would surround the plate.

The procedure described so far can efficiently detect line segments with different orientations. Figure 6 shows the process of food plate region detection. However, threshold selection may generate duplicated line segments (see Figure 6c). One possible solution to obtain the four boundary-line segments for the food plate would be to set to find the close line segments from an interior point toward the four different sides. The steps to leverage the closest four line segments surrounding the food plate and find the four corner points of the food plate are shown in Algorithm 1.
**Algorithm 1***Food plate detection**Input: an RGB image captured by Kinect* *Output: four corner points of the food plate* *1.*       *Calculate the center point p of the captured image. The point p would locate inside the food plate.* *2.*       *Draw a vertical ray along the y-axis with starting point p and upwards until it meets a red line. The red line L_1_ is selected as the upper edge of the plate. Calculate the red line’s slope and the angle θ between the line and x-axis.* *3.*       *Draw a parallel rightwards ray of L_1_ with starting point p until it meets a red line and denoted as L_2_.* *4.*       *Draw a parallel leftwards ray of L_1_ with starting point p until it meets a red line and denoted as L_3_.* *5.*       *Draw a vertical downwards ray of L_1_ with starting point p until it meets a red line and denoted as L_4_.* *6.*       *Determine the four corners from the extracted four lines L_1_ to L_4_ and redraw a rectangle (bounding box) and then output the four corner points.*

To obtain a plate normalized form, the mass center of the extracted food plate is calculated with the following equations. First, the food plate image moment is defined as $M_{ij}\;and$ $I\left(x,y\right)$ represents the image grayscale value in image coordinate (*x*,*y*).

(3) $$M_{ij}=\underset{x}{\sum }\underset{y}{\sum }x^{i}y^{j}I\left(x,y\right).$$

The plate image center $\left(c_{x},c_{y}\right)$ is defined as

(4) $$\left(c_{x},c_{y}\right)=\left(\frac{M_{10}}{M_{00}},\frac{M_{01}}{M_{00}}\right).$$

Finally, we can obtain a normalized image by performing a θ rotation with plate image center $\left(c_{x},c_{y}\right)$. The details about Equations (3) and (4) could be found in reference [19].

The process of traditional image recognition includes three consecutive steps, i.e., image segmentation, feature extraction, and image classification. A food image may contain multiple dishes (such as food in a buffet). Image cutting is to divide the image into different areas, and each area represents a type of dish (food item). If food image cutting is implemented correctly, it will help the accuracy of subsequent image feature extraction and image recognition and have an absolute impact on the calculation of food volume and nutrients. When the buffet dishes are arranged neatly (some dishes are separated by grids), a better cutting result can be obtained, that is, several dishes are cut into several nonoverlapping areas. 

**Figure 7:**
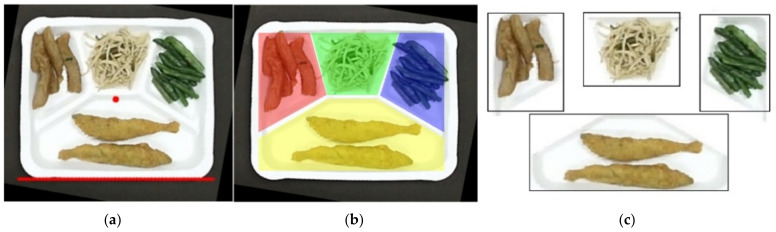
Plate normalized form and the segmentation of food items with a predefined mask: (**a**) the plate image center; (**b**) the four grids with different colors; (**c**) the segmented dishes.

In our experiments, the food plate is segmented into four regions with a predefined mask that is separated by grids. Figure 7b shows the mask containing four grids with four different colors. The food items on different regions in the plate can be easily segmented with this mask. Figure 7a,c shows the plate normalized form and the segmented dishes, respectively.

### 3.3. Food Image Recognition with Convolutional Neural Networks

Deep convolutional neural networks (DCNNs) have achieved state of the art in a variety of food image recognition tasks. However, there still exists no effective Chinese food recognition system matured enough to be used in the real world. The main reasons are the changeable factors including the lighting variation, scale variation, viewpoint variation, and background variation. In this study, we tried to control these variables in the proposed Bento buffet pricing system. Food recognition is a core process in the pricing system. The image dataset for food recognition is relatively small in our application since the number of dishes for a Bento buffet store is limited. In this study, many generally used CNNs were selected for food image recognition in the proposed application. Theses CNN models include AlexNet [12], VGG [20], ResNet [21], Inception [22], and DenseNet [23] and are explained below. The basic concepts about the deep-learning models could be found in the highly cited references [24,25].

#### 3.3.1. AlexNet

AlexNet [12] participated in the ImageNet Large Scale Visual Recognition Challenge (LSVRC) in 2012. The network’s top five error rate was 15.3%, which was 10.8% lower than the second place error rate. AlexNet contains eight layers; the first five layers are convolutional layers, some of which are maximum pooling layers, and the last three layers are fully connected layers. Nonsaturated ReLU activation function is used in this network, and its training performance has been improved, compared with tanh- and S-curves.

#### 3.3.2. VGG

VGGNet [20] can be regarded as a deepened version of AlexNet, which is composed of two parts—convolutional layer and fully connected layer. An improvement of VGG compared to AlexNet is to use consecutive 3 × 3 convolution kernels instead of larger ones (11 × 11, 7 × 7, 5 × 5). For a given receptive field (the local size of the input image related to the output), using a stacked small convolution kernel is better than using a large convolution kernel because multiple nonlinear layers can increase the depth of the network to ensure a more complex learning mode, and the cost is relatively small (fewer parameters). VGG16 contains a 16-layer architecture and VGG19 contains a 19-layer architecture. The input size of the network is 224 × 224.

#### 3.3.3. ResNet

From experience, the depth of the network is crucial to the performance of the model. When the number of network layers is increased, the network can extract more complex feature patterns, and therefore, theoretically better results can be achieved when the model is deeper. However, the experimental results found that the deep network has a degradation problem: when the network depth increases, the accuracy of the network becomes saturated or even decreases. The design of ResNet [21] has an architectural trick, which makes the depth of the network play a role. This trick is residual learning. ResNet101 is 101 layers deep. The input size of the network is 224 × 224. ResNet won the ILSVRC competition in 2015 with just a 3.6% error rate.

#### 3.3.4. Inception

Inception network [22] is an important milestone in the history of CNN classifier development. Before the advent of Inception, most popular CNNs simply stacked more and more convolutional layers to make the network deeper and deeper, hoping to achieve better performance. The Inception network is complex (requires a lot of engineering work). It uses a lot of tricks to improve performance, including speed and accuracy. Its continuous evolution has brought about the emergence of multiple Inception network versions. Inception v4 does not use the idea of residual learning and basically continues the structure of Inception v2/v3. 

#### 3.3.5. DenseNet

ResNet proves that deepening the number of network layers and improving the accuracy of the model can be taken into account through its unique design. DenseNet [23] uses a denser connection method. It is a dense convolutional neural network that uses a forward propagation method to connect each layer with the rest densely. The purpose of this network is to ensure that the information flow between the layers is maximized, and all layers (feature map size matching) are directly connected together. In traditional convolutional neural networks, the L layer will have a total of L connections, which is a one-to-one mode; in DenseNet, one layer will be connected to all other layers. Therefore, for the same L layer, there will be L(L + 1)/2 connections. This densely connected mode requires fewer parameters than traditional convolutional networks; hence, a densely connected mode can bring feature reuse, and there is no need to relearn redundant feature maps. Moreover, the operation of dimensional splicing brings rich feature information, and a lot of feature maps can be obtained with less convolution.

The setting of baseline CNN models is summarized and shown in Table 1. The input images are resized to 224 × 224 for AlexNet, VGG16, VGG19, ResNet50, and DenseNet121 architectures. On the other hand, the input images are resized to 299 × 299 for the Inception V4 architecture. 

**Figure 8:**
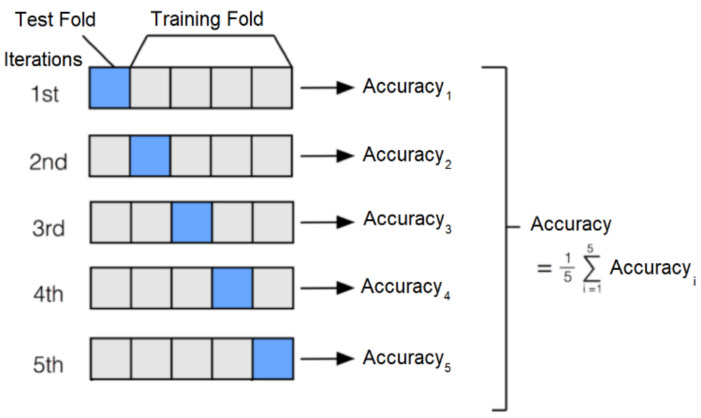
K-fold cross-validation method.

In order to be able to evaluate the stability of the above models more clearly, this research adopts the K-fold cross-validation method during training. Figure 8 shows the K-fold cross-validation method. The total dataset is divided into five equal parts, one for validation and the other four as a training set. A total of five rounds of training to ensure that each piece of data is tested as a validation set so that five validation accuracy rates can be obtained, and finally, they can be averaged as the overall performance of the model and then compared with other models.

### 3.4. Mapping from Color Coordinates to Depth Image with Kinect

Kinect has three lenses. The middle lens is an RGB color camera to collect color images. The left and right lenses are 3D structured light depth sensors composed of an infrared transmitter and an infrared complementary metal-oxide-semiconductor (CMOS) camera to collect depth data. There are two different coordinate systems—the depth camera coordinate frame of reference and the color camera coordinate system. In this study, the food volume is estimated by using the food area and the sum of corresponding depth values in the depth coordinate space. Therefore, a transformation from color space to depth space is required. 

**Figure 9:**
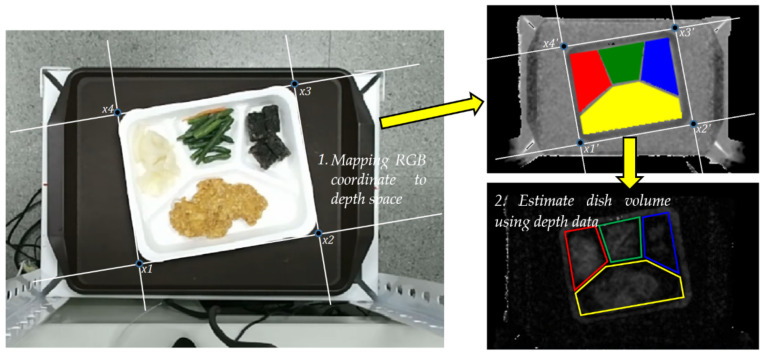
Mapping from RGB coordinate to depth coordinate and estimation of food volumes with depth values.

Figure 9 shows an example of mapping from RGB coordinate to depth coordinate and estimation of food volumes with depth values. When we want to map any sequence of 2D coordinate points $\left(x_{1},x_{2},x_{3},x_{4}\right)$ to another set of coordinate points $\left(x_{1}^{\prime },x_{2}^{\prime },x_{3}^{\prime },x_{4}^{\prime }\right)$, there must be at least eight conversion parameters between them. The projection transformation can be expressed as a linear mapping between the corresponding coordinates. Compared with the affine transformation, it has two more parameters $\left(a_{31},a_{32}\right)$ and can be written as

(5) $$\left(\begin{array}{c} {\overset{⌢}{x}}^{\prime }\\ {\overset{⌢}{y}}^{\prime }\\ h^{\prime } \end{array}\right)=\left(\begin{array}{c} h^{\prime }x^{\prime }\\ h^{\prime }y^{\prime }\\ h^{\prime } \end{array}\right)=\left(\begin{array}{ccc} a_{11} & a_{12} & a_{13}\\ a_{21} & a_{22} & a_{23}\\ a_{31} & a_{32} & 1 \end{array}\right)\cdot \left(\begin{array}{c} x\\ y\\ 1 \end{array}\right).$$

In Cartesian coordinates, the result of the mapping function is obviously nonlinear, and the conversion equation is as follows:

(6) $$x^{\prime }=\frac{1}{h^{\prime }}\cdot \left(a_{11}x+a_{12}y+a_{13}\right)=\frac{a_{11}x+a_{12}y+a_{13}}{a_{31}x+a_{32}y+1},\;y^{\prime }=\frac{1}{h^{\prime }}\cdot \left(a_{21}x+a_{22}y+a_{23}\right)=\frac{a_{21}x+a_{22}y+a_{23}}{a_{31}x+a_{32}y+1},$$

Projection transformation [26] is different from affine transformation [27]. A pair of parallel lines may not be parallel after being mapped, and the distance ratio between two points on the line will also change. The four corresponding two-dimensional coordinate points, $\left(x_{1},x_{2},x_{3},x_{4}\right)$,$\left(x_{1}^{\prime },x_{2}^{\prime },x_{3}^{\prime },x_{4}^{\prime }\right)$, and the points in the color image $x_{i}=(x_{i},y_{i})$ correspond to the points $x_{i}^{\prime }=(x_{i}^{\prime },y_{i}^{\prime })$ in the depth image, and the eight unknown conversion parameters can be solved by simply using linear equation solutions. After we bring the four points coordinates of the quadrilateral into Formula (3), we can obtain four sets of corresponding linear equations and eight conversion parameters $a_{11}\ldots a_{32}$ to be solved.

(7) $$\begin{array}{l} x_{i}^{\prime }=a_{11}x_{i}+a_{12}y_{i}+a_{13}-a_{31}x_{i}x_{i}^{\prime }-a_{32}y_{i}x_{i}^{\prime },\\ y_{i}^{\prime }=a_{21}x_{i}+a_{22}y_{i}+a_{23}-a_{31}x_{i}y_{i}^{\prime }-a_{32}y_{i}y_{i}^{\prime }, \end{array}$$

These eight parameters $a_{11},a_{12},\ldots ,a_{32}$ can be calculated using the four-point mapping method.

(8) $$a_{31}=\frac{\left(x_{1}^{\prime }-x_{2}^{\prime }+x_{3}^{\prime }-x_{4}^{\prime }\right)\times \left(y_{4}^{\prime }-y_{3}^{\prime }\right)-\left(y_{1}^{\prime }-y_{2}^{\prime }+y_{3}^{\prime }-y_{4}^{\prime }\right)\times \left(x_{4}^{\prime }-x_{3}^{\prime }\right)}{\left(x_{2}^{\prime }-x_{3}^{\prime }\right)\times \left(y_{4}^{\prime }-y_{3}^{\prime }\right)-\left(x_{4}^{\prime }-x_{3}^{\prime }\right)\times \left(y_{2}^{\prime }-y_{3}^{\prime }\right)}\;\;a_{31}=\frac{\left(x_{1}^{\prime }-x_{2}^{\prime }+x_{3}^{\prime }-x_{4}^{\prime }\right)\times \left(y_{4}^{\prime }-y_{3}^{\prime }\right)-\left(y_{1}^{\prime }-y_{2}^{\prime }+y_{3}^{\prime }-y_{4}^{\prime }\right)\times \left(x_{4}^{\prime }-x_{3}^{\prime }\right)}{\left(x_{2}^{\prime }-x_{3}^{\prime }\right)\times \left(y_{4}^{\prime }-y_{3}^{\prime }\right)-\left(x_{4}^{\prime }-x_{3}^{\prime }\right)\times \left(y_{2}^{\prime }-y_{3}^{\prime }\right)}\;\;{\;a}_{32}=\frac{\left(y_{1}^{\prime }-y_{2}^{\prime }+y_{3}^{\prime }-y_{4}^{\prime }\right)\times \left(x_{2}^{\prime }-x_{3}^{\prime }\right)-\left(x_{1}^{\prime }-x_{2}^{\prime }+x_{3}^{\prime }-x_{4}^{\prime }\right)\times \left(y_{2}^{\prime }-y_{3}^{\prime }\right)}{\left(x_{2}^{\prime }-x_{3}^{\prime }\right)\times \left(y_{4}^{\prime }-y_{3}^{\prime }\right)-\left(x_{4}^{\prime }-x_{3}^{\prime }\right)\times \left(y_{2}^{\prime }-y_{3}^{\prime }\right)}\;a_{11}=x_{2}^{\prime }-x_{1}^{\prime }+a_{31}{\times x}_{2}^{\prime }\;a_{12}=x_{4}^{\prime }-x_{1}^{\prime }+a_{32}{\times x}_{4}^{\prime }\;a_{13}=x_{1}^{\prime \;}\;a_{21}=y_{2}^{\prime }-y_{1}^{\prime }+a_{31}{\times y}_{2}^{\prime }\;a_{22}=y_{4}^{\prime }-y_{1}^{\prime }+a_{32}{\times y}_{4}^{\prime }\;a_{23}=y_{1}^{\prime }$$

Once the food in the image is identified, if the volume of the food can be further estimated, the corresponding price can be calculated based on the volume. The nutrition assessment app proposed by Pouladzadeh et al. [28] uses fingers as a reference to calculate the actual area and height of the food in the top view image and the side image and then estimate the volume of the food. However, the above method is limited due to the need to use reference objects; in this paper, we used a depth camera to obtain the depth information of the food and further estimated the volume of the food. 

Initially, the average depth value of a region A in an empty plate is estimated and denoted as $d_{empty}\left(A\right)$. Image-based food volume evaluation must identify the area occupied by all food items, perform image cutting of the food objects in the image, and then calculate the volume of each segmentation item. In this way, the volume of a certain food on *A* is calculated, as long as the depth values of all the pixels in the cutting area of the food on region *A* are summed up. The volume of the food can be estimated and is denoted as

(9) $$\mathrm{Volume}\left({\mathrm{food}}_{A}\right)=\alpha \times \sum \left|d\left(x_{i},y_{i}\right)-d_{empty}\left(A\right)\right|,\;\left(x_{i},y_{i}\right)\in A,$$

where $d\left(x_{i},y_{i}\right)$ represents the depth value with food on the plate at the image coordinates $\left(x_{i},y_{i}\right)$ and α is a calibration parameter. In order to measure the correlation between the volume estimation using Equation (9) and the actual food volume, we selected different food items for testing. The experiments used Archimedes’ law to calculate the actual volume of a food object by measuring the amount of water rising when the food was put into the water of a measuring cup. Finally, α value was estimated as 1.2 from a linear regression result. 

**Figure 10:**
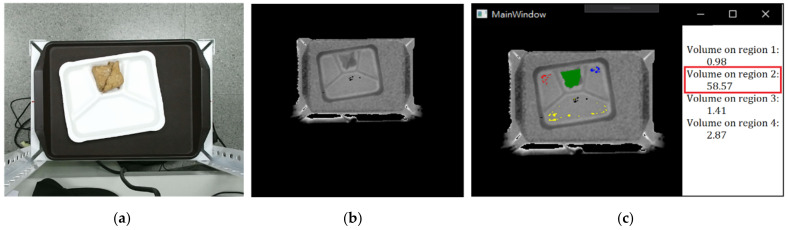
Food volume estimation. (**a**) RGB image, (**b**) the corresponding depth image, and (**c**) the estimated volumes on different regions.

In practical applications, we set up a food volume threshold for each food per unit. If the food volume on A is larger than the threshold, double times of money is asked to pay. Figure 10 shows an example of volume estimation using a depth image for oily bean tofu. The estimated volume of tofu in region 2 is 58.57, and the volumes of other regions are relatively small, meaning no food in these regions.

## 4. Experiments Results and Discussions

### 4.1. Experimental Environment Setting

**Figure 11:**
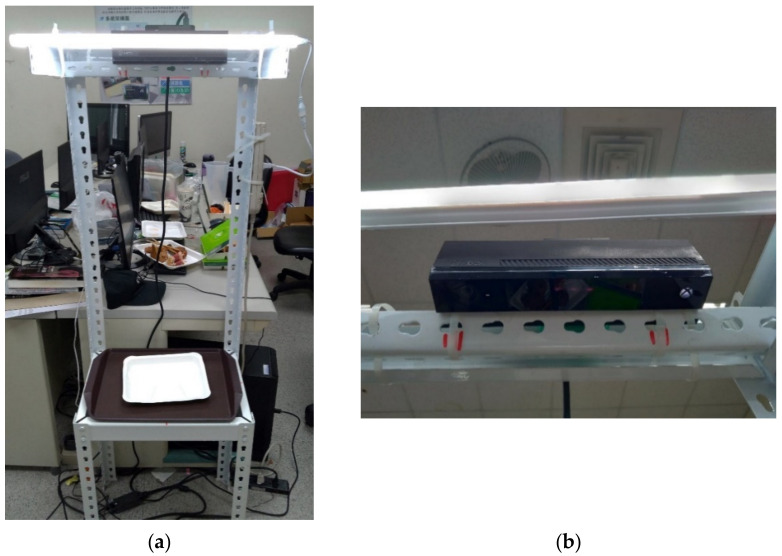
The proposed system prototype of food recognition for the Bento buffet: (**a**) the prototype contains an angle steel shelf, a Kinect camera, a light source, and a desktop computer; (**b**) Kinect Xbox One sensor.

When performing image recognition, light can easily affect the recognition result. Therefore, this system sets up a LED lamp on the top to ensure the consistency of light, as shown in the upper part of Figure 11a. The Kinect is installed next to the lamp, and the camera is vertically downward to shoot, as shown in Figure 11b. 

The proposed method contains two stages—the training and test stage. In the training stage, the DGX-1 machine with high-speed graphics processing unit (GPU) computations was used to training CNN models from scratch for our food image dataset and labels. In the test stage, the workstation computer running the client–server architecture of this proposed system to recognize the test images with the well-trained CNN models. The environment contents of hardware and software, which are described and shown in Table 2. In the training stage, we set the initial learning rate as 0.001. The number of epoch for the training is set to 100. Stochastic gradient descent (SDG) with momentum was selected as the training optimization method in our experiments. 

### 4.2. Food Image Dataset

**Figure 12:**
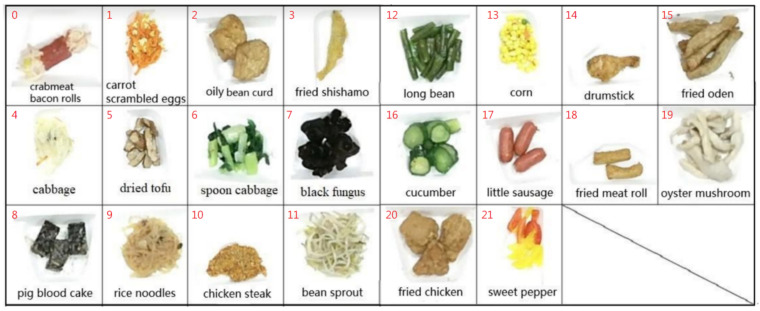
The list of 22 different food items with id numbers in a Bento buffet cafeteria in our experiments.

In the process of building a food image dataset, the food plate with food items was put on a black tray of the steel shelf. The lighting condition was uniformly adjusted, and then the captured image of the tray was saved in the storage. Then, the individual food images were cut out from the captured images. Generally, the number of food is less than 30 in a Bento buffet cafeteria in Taiwan. Our food source comes from a school Bento buffet cafeteria. Two datasets were collected for the experiments in the training process and testing process, respectively. The training dataset has a total of 22 classes, and each class contains about 90 to 95 samples. In total, there are 2025 original samples, 1613 training samples, and 412 validation samples. These image samples are used for our experiments in the training process. On the other hand, we have made another image shooting on food items for the testing samples. The testing dataset has the same 22 classes of images as the training dataset, and each class contains 10 samples. In total, there are 220 test samples and, these images are used for the experiments in the testing process. Figure 12 shows the 22 different food items in the Bento buffet cafeteria for our experiments.

### 4.3. Experimental Results and a Hierarchical Approach 

In the experiments of the training process, we tested the performance of six different CNN models: AlexNet, VGG16, VGG19, ResNet-50, Inception-v4, and DenseNet-121. The images in each class are divided into five folds, and four folds were used for training, while the remaining one fold was used for validation. A five-fold cross-validation method was applied in our experiments, and the validation accuracy results could be found in Table 3. From the results, we can infer that Inception-v4 obtained the highest validation accuracy rate than others in the training process. However, it needs the most time (8649 s) to train the model using the training dataset. AlexNet model requires the least execution time (1226 s) to train the model using the training dataset.

In the testing process, the six well-trained models generated in the training process are used on the testing dataset to evaluate the food image recognition performances. The experimental results are shown in Table 4. ResNet model obtains the best recognition rate of 0.986 on the testing dataset. AlexNet model requires an average of 0.062 s to execute the recognition for an image. 

**Figure 13:**
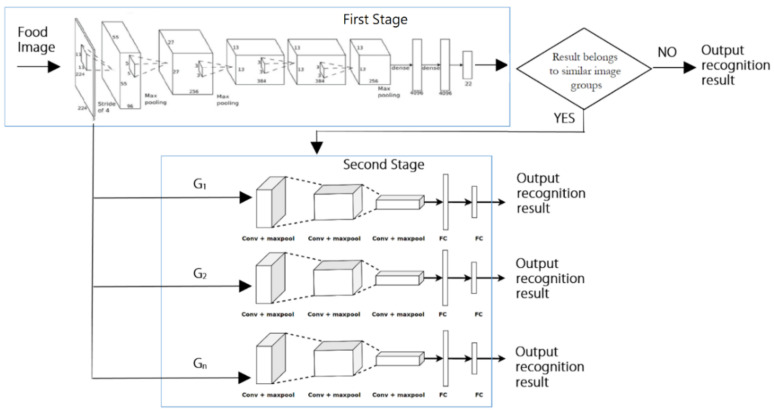
The overall architecture of the proposed two-stage hierarchical approach to enhance the recognition rate.

The purpose of designing the checkout system is to reduce the waiting time for customers to queue for checkout. Therefore, it is hoped to have a fast visual checkout, and the recognition time for dishes on the food plate should be limited (i.e., under 1 s). Therefore, we choose AlexNet as our basic model. Although the recognition rate of AlexNet is not the highest, the execution time is the best. Then, a hierarchical CNN structure that combined the AlexNet network in the first stage and a set of shallow CNN models in the second stage is proposed. The proposed approach can be regarded as a two-stage classifier that can recognize food images from coarse to fine. Figure 13 presents the overall architecture of the proposed structure. It is designed based on the AlexNet network in the first stage using the training dataset. The recognition rate of AlexNet can only achieve a 94.5% accuracy for all test images because some similar images exist in the dataset. The prediction probabilities of these images are relatively low. Therefore, we extract similar images to perform the further classification in the second stage.

**Figure 14:**
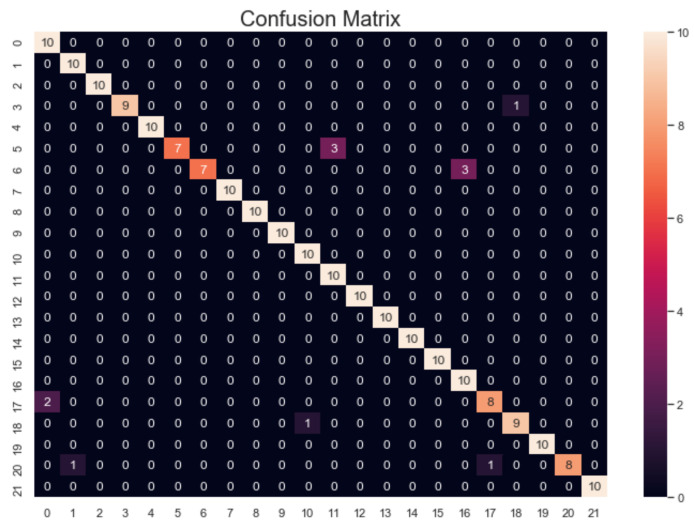
The confusion matrix of recognition result using the AlexNet model on the test image dataset.

**Figure 15:**
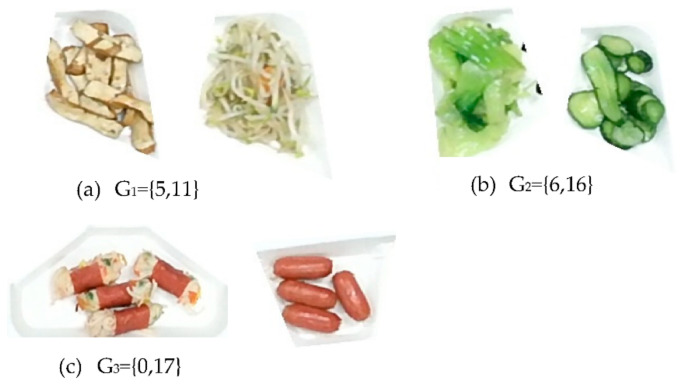
Three similar image groups were found out from the confusion matrix.

Similar food image pairs were extracted based on the first stage results. In our case, the confusion matrix of the first stage result on the test dataset is shown in Figure 14. For the image recognition result of test images in class I, if more than α samples are mistakenly identified to another class j, then class I and class j will be defined as a similar group. For example, there are three similar image groups in the confusion matrix in Figure 15 if α = 1: G_1_ = {5,11}, G_2_ = {6,16} and G_3_ = {0,17}. Where {x, y} means that class x and class y belong to a similar group. The image pairs of G_1_, G_2,_ and G_3_ are shown in Figure 14. The color distributions of the images in each image pair look very similar, and the AlexNet model cannot identify them very well.

**Figure 16:**
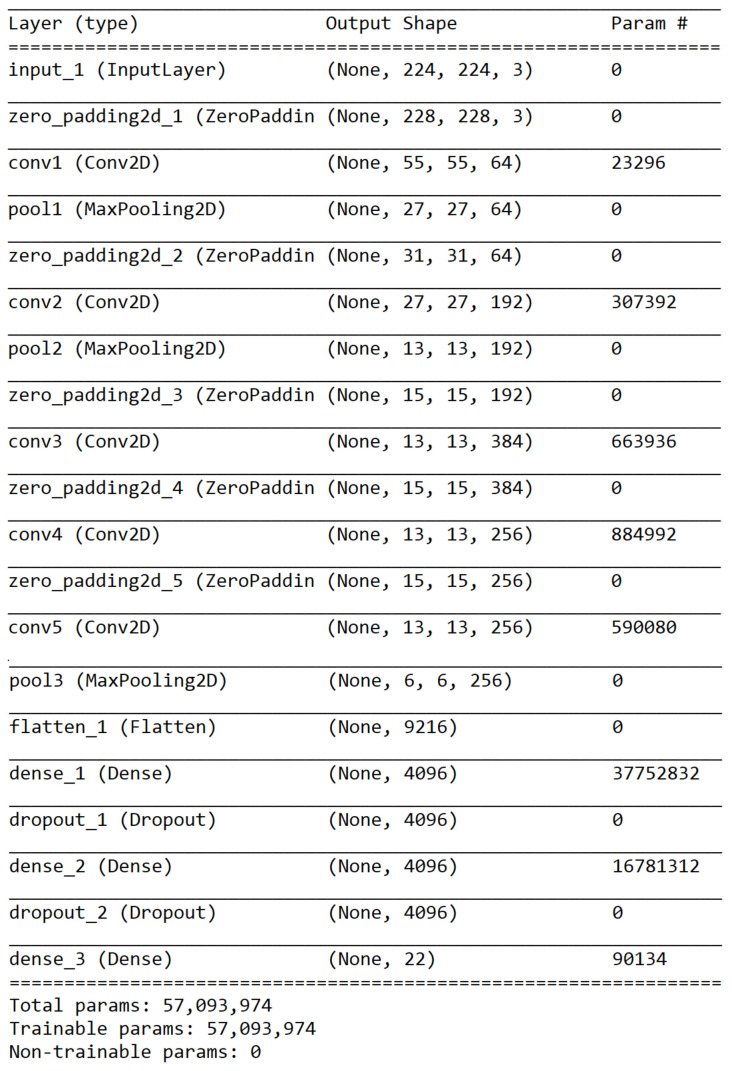
A summary of the AlexNet model in the first stage of the proposed method.

**Figure 17:**
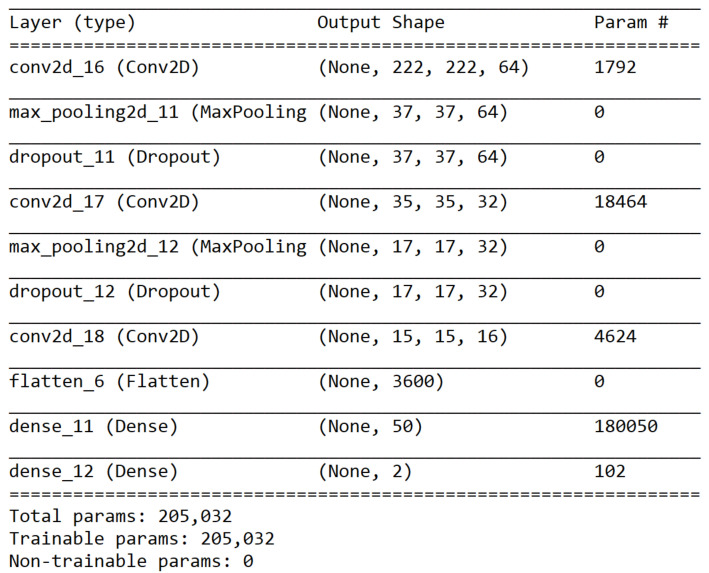
A summary of the CNN model in the second stage of the proposed method.

**Figure 18:**
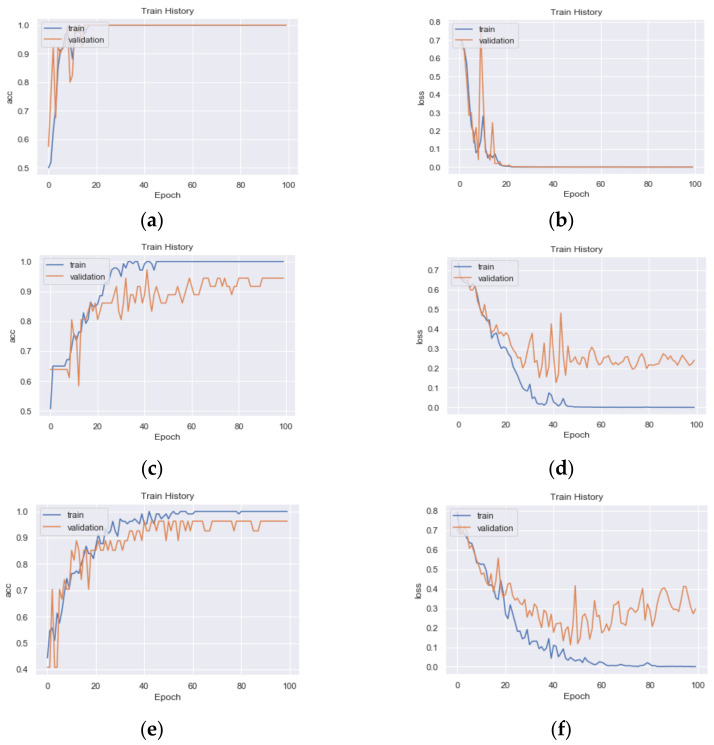
The learning of CNN models for G1, G2, and G3: (**a**) training accuracy vs. validation accuracy for G1; (**b**) training loss vs. validation loss for G1; (**c**) training accuracy vs. validation accuracy for G2; (**d**) training loss vs. validation loss for G2; (**e**) training accuracy vs. validation accuracy for G3; (**f**) training loss vs. validation loss for G3.

AlexNet model in the first stage of the proposed method is critical to the second stage’s performance. The configuration of the AlexNet model is shown in Figure 16. The first stage was trained on 22 classes using the training dataset. Similar image groups were extracted from the result of the first stage. These groups were then selected to train the second stage. For each similar group, a three-layer CNN model was trained using the corresponding images in the training dataset. The configuration of the three-layer CNN model is shown in Figure 17. The training statuses of CNN models for G1, G2, and G3 are shown in Figure 18, respectively. We can find that the validation accuracies on the curves are very high. 

**Figure 19:**
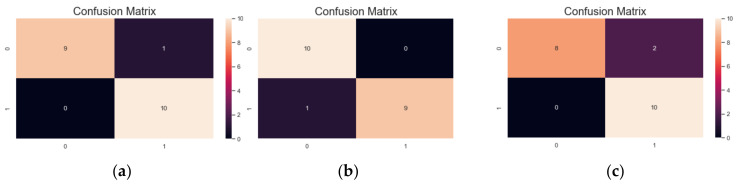
The confusion matrices of recognition results using a three-layer CNN model on the test images belong to similar image groups: (**a**) G1, (**b**) G2, and (**c**) G3.

To evaluate the performance of proposed two-stage hierarchical approach, the images in the testing dataset were applied. In the first stage, the AlexNet model will perform the recognition result and output the predicted class for each test image except if the predicted class belongs to the three similar image groups G1, G2, and G3. In the experiment, there are 20 sample images for every group that will be sent to the second stage for further recognition. The recognition results for these images in G1, G2, and G3 are shown as the corresponding confusion matrices in Figure 19.

In Figure 13, we can find eight misidentified test images belonging to the three similar groups using the AlexNet model. Now we can reduce the misidentified number to four using the two-stage approach. The accuracy of the proposed approach reaches 96.3%, which is an increase of 1.8%. The execution time overhead of the proposed method is very light since the image recognition in the second stage requires only 0.046 s for a test image. Therefore, the total execution time to recognize a test image using the proposed two-stage method requires 0.108 s. This speed performance plays a crucial factor in the design of an automatic checkout system for Bento Buffet. The test accuracy using different CNN models and the proposed method on the testing dataset are compared and shown in Table 4.

In our application, we choose AlexNet as the baseline network model for the first stage. The recognition rate of AlexNet in this application is already very high. The work of the three-layer convolutional neural network in the second stage is mainly to identify poorly similar images in the first stage. Although the proposed architecture requires a two-stage identification, the convolutional neural networks used in the first and second stages both have the characteristics of a small number of layers and less memory, which can reduce the time complexity of execution.

**Figure 20:**
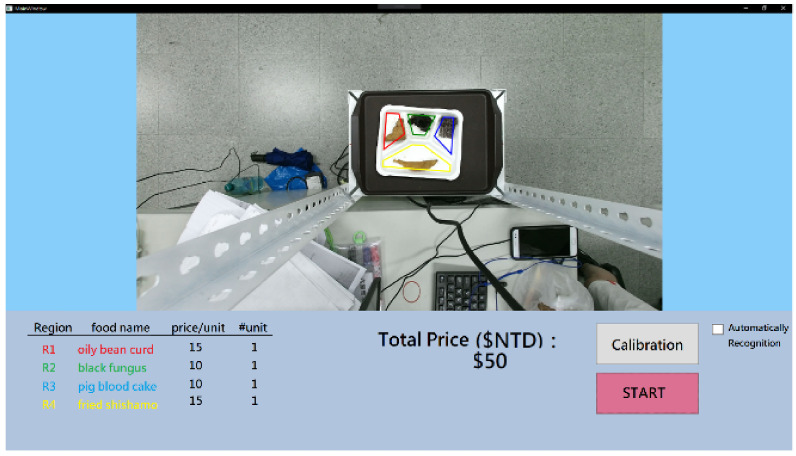
An example using the proposed visual checkout system to estimate food price.

**Figure 21:**
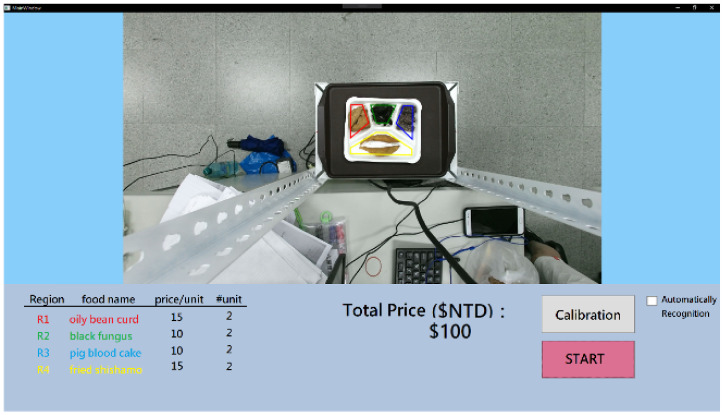
An example using the proposed visual checkout system to estimate food price.

Finally, we designed a user interface of the visual checkout system for Bento Buffet restaurants. Figure 20 and Figure 21 show two examples with the same food items on the plate using the proposed checkout system. The food volume in Figure 20 is larger and the estimated price is double than the other.

## 5. Conclusions

In this paper, an AI visual checkout system for a Bento buffet was designed and implemented. The system has a high possibility to reduce the customer waiting time effectively and minimize the labor cost for Bento buffet cafeterias. We used computer vision and deep-learning technology to design and implement the system. The prototype contains an angle steel shelf, a Kinect camera, a light source, and a desktop computer. Convolutional neural networks including AlexNet, VGG, ResNet, DenseNet, and Inception v4 were applied for food recognition with an augmented food image dataset. There are 22 different food items included in the dataset from a Bento buffet cafeteria. The contribution of this paper is outlined as follows:

(1) We presented a new framework of visual checkout system for Bento buffet cafeterias to help reduce waiting time in the queue for customers;
(2) We presented a food plate detection method using Hough transformation, and it could automatically capture and segment the food images from the food plate;
(3) We presented a hierarchical approach with two stages, and this approach performed remarkably in both the recognition accuracy rate and the training and recognition time;
(4) We presented a simple food volume estimation method using the corresponding depth image.

## Figures and Tables

**Table 1:** The baseline convolutional neural network (CNN) model settings.

Model	Input Image Shape	Layers	Parameters
AlexNet	224 × 224 × 3	8	62,378,344
VGG16	224 × 224 × 3	16	138,357,544
VGG19	224 × 224 × 3	19	143,667,240
ResNet50	224 × 224 × 3	50	25,636,712
Inception-v4	299 × 299 × 3	48	41,303,274
DenseNet121	224 × 224 × 3	121	8,062,504

**Table 2:** The environment contents of hardware and software in our experiments.

Hardware		
	NVIDIA DGX-1	Workstation
OS	Ubuntu 16.04	Windows 10 Professional
CPU	2×Intel Xeon E5-2698 v4 2.2 GHz	Intel^®^ Xeon^®^ E5-1650 v4 3.60 GHz
RAM	512 GB DDR4 2133	16 GB
GPU	8×Tesla P100	Nvidia RTX 2060
**Software**		
	Client	Web Server
Programming Language	C#	Python
Development Tool	Visual Studio 2019	Sublime
Framework	NET 4.7.2	Flask 1.1.1
Packages	Kinect SDK v2	OpenCV 4.1.0.25 Keras 2.2.4

**Table 3:** Validation accuracy with different CNN models on training image dataset.

Recognition Rates of Different Deep Networks on Our Original Food Dataset							
	1^st^ Run	2^nd^ Run	3^rd^ Run	4^th^ Run	5^th^ Run	Average Test Accuracy	Average Training Time(s)
AlexNet	85.19	89.26	84.97	89.02	84.09	86.51 ± 2.1	1226
VGG16	84.95	84.87	79.55	82.29	83.83	83.1 ± 2	2497
VGG19	70.14	82.68	78.57	72.06	76.51	75.99 ± 4.5	2889
ResNet-50	86.4	97.07	97.78	88.27	83.83	90.67 ± 5.6	2762
Inception-v4	99.27	99.26	98.52	99.75	98.73	99.11 ± 0.4	8649
DenseNet-121	98.54	99.02	98.52	98	96.21	98.06 ± 0.9	5956

**Table 4:** Test accuracy with different CNN models and proposed method on the testing dataset.

CNN Model	Test Accuracy	Average Recognition Time Per Image(Second)
AlexNet	0.945	0.062
VGG16	0.875	0.65
VGG19	0.909	1.04
ResNet50	0.986	1.34
Inception v4	0.877	3.04
DenseNet121	0.945	2.02
Proposed	0.963	0.108

## Data Availability

Not applicable.
